# Adaptation and Validation of a Questionnaire to Evaluate Knowledge of the Low Phe Diet in PKU

**DOI:** 10.3390/nu13082719

**Published:** 2021-08-07

**Authors:** Rodolfo Ramos-Álvarez, Maili Kapp, María Mercedes Rodríguez-Ruiz, Rocío Fausor, María Amor Bueno-Delgado, Kirsten Ahring, Susan E. Waisbren

**Affiliations:** 1 Department of Social Psychology, Melilla Campus, Granada University, 52005 Melilla, Spain; 2 Teaching Innovation Project (Code: 14–82), Melilla Campus, Granada University, 52005 Melilla, Spain; mailikapp@correo.ugr.es; 3 Osakidetza, Basque Health Service, 010006 Vitoria, Spain; rrmertxe@gmail.com; 4 Department of Personality, Assessment and Clinical Psychology, Complutense University of Madrid, 28223 Madrid, Spain; rociofausor@ucm.es; 5 Pediatrics Service, Virgen del Rocío University Hospital, 41013 Sevilla, Spain; mabuenod@gmail.com; 6 Center for PKU, Copenhagen University Hospital, 2600 Copenhagen, Denmark; kirsten.ahring@regionh.dk; 7 Genetics and Metabolism Programs, Boston Children’s Hospital, Harvard Medical School, Harvard University, Boston, MA 02115, USA

**Keywords:** phenylketonuria, diet knowledge, PKU, validation and adaptation of questionnaire

## Abstract

Phenylketonuria (PKU) is an autosomal recessive disorder of phenylalanine (Phe) metabolism, causing a build-up of Phe in the body. Treatment consists of a Phe-restricted diet for life and regular determination of blood Phe levels to monitor the intake of Phe. Despite the fact that diet is the cornerstone of treatment, there are no studies examining common knowledge about food items and whether they are allowed as part of the PKU diet. Improving parents’ and patients’ knowledge and competence about the diet enables them to make appropriate food choices. This study validates a food-knowledge questionnaire first developed in Spanish and modified for English speaking populations. The questionnaire potentially helps parents to prepare appropriate meals and healthcare providers to create individualized educational programs about PKU for children and adolescents with this disorder.

## 1. Introduction

Phenylketonuria (PKU), or phenylalanine hydroxylase deficiency (PAH deficiency), is an autosomal recessive disorder of phenylalanine (Phe) metabolism caused by a deficiency of the enzyme phenylalanine hydroxylase (PAH). Phe is one of the essential amino acids found in all protein. The inability to convert Phe to tyrosine causes the accumulation of Phe in the body. Excess Phe is toxic to the central nervous system [1,2]. The prevalence varies between countries. It is estimated to be 1:4000 in Turkey, 1:10,000 in Europe and between 1 in 10,000 to 15,000 in children born in the United States [3,4].

Before treatment, three phenotypes are differentiated based on blood Phe concentrations measured prior to initiating treatment. (1) In classic or severe PKU, Phe values exceed 1200 µmol/L. Untreated severe PKU leads to permanent intellectual disability, seizures, developmental delay, behavioral problems, and psychiatric disorders [5,6]. (2) In mild PKU, blood Phe concentrations are between 600–1200 µmol/L. Most centers now recommend dietary treatment and/or treatment with sapropterin dihydrochloride (BH4) for infants and children with blood Phe levels within this range. BH4 is a co-factor of the deficient enzyme and when supplemented is often effective in reducing blood Phe concentrations in mild PKU. (3) Mild hyperphenylalaninemia, with blood Phe concentrations 120–600 µmol/L, may not require dietary treatment [4,7,8].

There is consensus on the importance of controlling Phe in the blood, as measured through restricting dietary Phe [7,9]. Adherence to diet has been shown to be a reliable predictor of clinical outcomes. Frequent blood tests are required to assess metabolic status and determine an adequate dietary intake of Phe. In order to meet the body’s requirements for protein, individuals with PKU are prescribed a protein substitute with all the necessary nutrients and amino acids in protein apart from the “offending” amino acid, Phe. It also contains the amounts of fat, carbohydrate and micronutrients suitable or right for patients of a particular age. They consume this protein substitute, called a medical food, at least three times a day. It prevents the deficiency that may result from a limited intake of other amino acids and variable amounts of carbohydrates, fats, vitamins, and minerals [6,9,10].

Adhering to the Phe restricted diet for life is essential [10]. Initially, children with PKU will be guided by parents and later in adolescence and adulthood by self-management. This reduces the risks of cognitive decline in babies and mental and neurological problems in adults [11,12]. However, a low Phe diet during childhood but abandoned later in life can increase the risk of other adverse outcomes, such as depression, anxiety and executive functioning deficits in adulthood [13].

During adolescence and adulthood, many individuals with PKU find that the limited diet is unpleasant, difficult to follow and a hindrance to social relationships [14,15]. Studies show that PKU diet adherence decreases with age [16,17,18] and many patients are lost to follow-up [19]. Many factors can influence eating behavior. Different studies have considered demographic factors [20,21,22], educational level [23], environmental factors [15], nutritional knowledge and parents’ attitudes [24,25] and psychosocial factors [21], among others.

Equally important is knowledge and competence about diet, enabling the correct food choices [24,25,26]. Parents and individuals with PKU need to understand the age-appropriate nutrition recommendations as well as food-based guidelines, and how to apply these to food choices and eating behaviors [27,28].

Witalis and colleagues reported that only 33 to 40% of patients with PKU (10–19 years and >20 years) knew the Phe content in the most common food products [29]. Other authors have found that the parents’ poor dietary knowledge may have an impact on long-term metabolic control in children [30]. Some authors observe a negative correlation between maternal knowledge about food and the average concentration of Phe in the blood in the child [31]. In the general population, consumers from different countries vary in their level of knowledge about nutrition [27].

The early detection of misinformation and knowledge gaps among parents and patients would alert caregivers to provide training and to focus education on specific areas needed to improve motivation, dietary management, and adherence to medical recommendations [19,32].

Despite the fact that diet is the cornerstone of treatment, there are no objective measures of knowledge in English related to the allowed and disallowed foods in PKU.

The objective of this study was to adapt a Phe diet questionnaire, originally developed in Spain, for individuals with PKU and their parents. The questionnaire was translated into English and modified to incorporate foods commonly found in the United States (USA). Caregivers and individuals with PKU in the USA completed the questionnaire, representing the first of this kind to be administered by healthcare professionals.

## 2. Methods

### 2.1. Description of the Spanish Questionnaire

The Spanish questionnaire “Cuestionario de valoración de conocimientos sobre la dieta baja en fenilalanina en familiares de afectados por la fenilcetonuria” (Questionnaire to evaluate knowledge of the low Phe diet in PKU) provided the template for the questionnaire developed for the English speaking population in the USA. For the Spanish version, a representative sample of items taken from a list of all food types was chosen. Food selection for this representative sample was based on the following criteria or guidelines:

1. The amount of Phe in a food determined whether it was prohibited or allowed. The limit was set at 50 mg of Phe per 100 g product.
2. The proportion of prohibited and permitted foods included in the questionnaire was the same, with 34 items of permitted foods (42.5%) and 46 of prohibited foods or allowed only in very small quantities (57.5%).
3. All food categories were included: meat, fish, seafood, eggs, dairy, cereals, legumes, vegetables, fruits, beverages, sauces, sweets and condiments.
4. Within the different food categories, there were regularly consumed foods (prototypical) and less frequently consumed foods (non-prototypical).
5. All foods in the list were easily available and accessible for consumption.

The final Spanish version of the questionnaire included 80 items of food to be rated as “allowed” or “prohibited”. In addition, there were questions to elicit basic health and demographic information (age, sex, town of residence, level of education and type of phenylketonuria) and information about the person completing the questionnaire (age, sex, town of residence, level of education and relationship to the individual with PKU). The Spanish version of the questionnaire was found to be psychometrically sound, with a Cronbach’s index of 0.87. The Spanish version is available from the first author (R.R.-Á.) and a manuscript describing its development is forthcoming.

### 2.2. Translation and Adaptation of the Spanish Version to American Population

The Spanish questionnaire was translated into English and adapted to incorporate contemporary food practices and colloquial or common language. Guidelines proposed by Muñiz and Hambleton (1996) and Muñiz, Elosua and Hambleton (2013) were used [33,34]. After carrying out a morpho-semantic assessment, additional modifications about sociodemographic questions were made. Finally, items that presented comprehension problems were rewritten. A multinational and multidisciplinary panel of experts and consumers participated in the item selection phase to establish the questionnaire’s content validity. The panel included nutrition professionals, physicians, and other healthcare providers, as well as affected individuals and their parents recruited from the National PKU Alliance and the California PKU Association. In addition, a group of 6 native English speakers from outside of the PKU community, reviewed the questionnaire and provided suggestions regarding wording and item selection.

The questionnaire adaptation to American English revealed some differences related to expressions, the use of some foods and words to define them. For example, “Identification of the surveyed family member” was changed to “Data about the person who completes this survey.” Items related to education were also changed: “Nursery” became “kindergarten”; and “Number of years of education” replaced “Your level of education.” The most frequent changes related to food. For example, “Horchata” (a typical refreshing drink from an area of Spain) is almost unknown. The words “sweet potato” and “yam” led to confusion since the words are often used interchangeably in the USA, although referring to different species of tuberous vegetables. Lentils, commonly eaten in Europe, were not recommended until 2010 in the Dietary Guide for Americans and were dropped from the list. “Seasoning” replaced “vegetable concentrate” and “lobster” and “shrimp” were included instead of “prawns”.

Two versions of the questionnaire were made: a computer version and a paper version. Both versions were intended to be administered by a health professional but completed as a self-report questionnaire. Analyses revealed that both were assessing the same construct and that the data collection by the two different systems (paper and computer) did not affect the results. A forthcoming manuscript details the statistical methods used in the development of the questionnaire.

After initial piloting, additional terms were changed if found to be confusing, ambiguous or related to food items not in the diet. The final questionnaire (Supplementary Materials) included 80 items adapted to the American population. In addition, brief instructions were written. As the questionnaire involved simply placing a checkmark next to each food item, almost all participants completed the task in under 10 min, including completion of the sociodemographic questions.

### 2.3. Statistical Analyses

Once the translation was verified and content validity was determined, statistical analyses were performed to address psychometric properties of the questionnaire. A factor analysis was carried out with the Factor program (Tarragona, Spain) (Lorenzo-Seva and Ferrando, 2013) [35]. A tetrachoric correlation matrix, ULS fit and Promin rotation were used. Calculations were made for determining a non-normalized fit index (Tucker and Lewis, 1973) [36], a comparative fit index, a goodness of fit index and adjusted goodness of fit index. Details regarding the more extensive statistical methods to document the psychometric properties of the questionnaire will be included in a forthcoming manuscript.

Discrimination: item-total correlations were verified to identify the extent to which answering an item correctly was related to scoring high on the entire subscale. Items with Chronbach Alpha values below 0.30 would be eliminated.

Mean and variance: the questionnaire represents optimal performance (right/wrong responses). Therefore, items with extreme mean difficulty values (either too easy or too hard) were eliminated. Items with mean difficulty values close to 0.5 would be retained. Similarly, items that presented a very small variance, close to 0, would also be eliminated. Data were processed using IBM SPSS Statistics 25 software (Armonk, NY, USA). The level of statistical significance was set at *p* < 0.05.

### 2.4. Ethical Considerations

The research was carried out following recommended standards for research on human participants of the European Community Code of Ethics as well as Ethical Research and Publication Standards of the Psychological Association. The research was approved by the vice-rector for research of the UGR and the Melilla Campus, Granada University, Granada Spain, with the approval of the National PKU Alliance and the California PKU Association. Privacy in data processing was guaranteed. Participation in the study was voluntary and anonymous.

## 3. Results

### 3.1. Descriptive Analysis

The questionnaire was completed by 177 parents of children with PKU living in the USA. The mean age of the parents was 40.31 (SD = 8.66), with a range of 24–76 years. The mean age of their children (*n* = 177) was 6.92 (SD = 4.77) ranging from 1 to 18 years. The demographic data of the sample are presented in Table 1.

According to follow-up assessments by the respondents to both the on-line and paper versions, the questionnaire was clear and easy to understand. They described the instructions and the response options as simple and free of ambiguity. They reported that they had no difficulty in choosing the answers.

### 3.2. Psychometric Properties of the Subscales

- Reliability analysis (internal consistency): global reliability was analyzed and confirmed. Without eliminating any of the final 80 items, Cronbach’s alpha coefficient was 0.945, indicating high internal reliability.
- Factor analysis: as described below, a satisfactory 3-factor factorial solution was revealed by parallel analysis using Factor program (Lorenzo-Seva and Ferrando, 2013) [35].
  Factor 1: foods allowed (factor reliability = 0.97).
  Factor 2: prohibited foods, easy to recognize (factor reliability = 0.92).
  Factor 3: limited foods, NOT easy to recognize (factor reliability = 0.91).

As shown in Table 2, the overall proportion of variance explained was 56%; 35% corresponding to factor 1, 14% to factor 2 and 7% to factor 3. The fit indices are shown in Table 3.

## 4. Discussion

Expert review and psychometric analyses support the use of a translated and adapted version of the questionnaire to evaluate knowledge of the low Phe diet in PKU. The questionnaire was designed to identify misinformation and gaps in knowledge regarding allowed and prohibited foods for individuals required to follow a diet for PKU. The results from the questionnaire provide a tool for dieticians and other healthcare providers advising parents and patients with PKU.

In addition, the questionnaire can be used in conjunction with new technologies, such as mobile apps or PKU-related games that can extend such training and permit access to materials and resources beyond specific advice. The questionnaire is quick to administer, easy to understand, and reliable. This is the first questionnaire designed for this purpose to be administered by healthcare professionals.

Participants in this study were not recruited based on the phenotype of the child with PKU. Thus, some individuals with milder phenotypes may be correct in identifying some foods with relatively high Phe content as allowed. However, the questionnaire includes an item regarding type of PKU so that individual results can be interpreted with this information in mind. The adaptation of the questionnaire to American English revealed some differences related to expressions, the use of some foods and words to define them. Thus, when used with individuals from other countries or for whom English is not a first language, some items may need to be explained or interpreted. In general, though, study participants accepted the questionnaire and found it easy to understand and complete.

The questionnaire is now available in translated and adapted versions in the US and Spain and may soon be available in Denmark and Estonia. Future research including cross-cultural studies correlating questionnaire results and indices of metabolic control will add to the understanding of factors related to dietary adherence in PKU.

## Figures and Tables

**Table 1:** Demographic characteristics of the participants.

		Online	Paper	Total
	Total participants *n* (%)	153 (86.45)	24 (13.55)	177 (100)
	Gender n (%)			
	Males	16 (10.46)	6 (25.0)	22 (12.43)
	Females	137 (89.54)	18 (75.0)	155 (87.57)
	Level of education *n* (%) Primary school			
		5 (3.26)	0 (0)	5 (2.82)
	Secondary school	18 (11.77)	1 (4.17)	19 (10.74)
Parents	College/University	130 (84.97)	23 (95.83)	153 (86.44)
	Relationship to the PKU			
	Child n (%)			
	No answer	0 (0)	1 (4.16)	1 (0.56)
	Father	13 (8.5)	3 (12.5)	16 (9.03)
	Mother	131 (85.62)	17 (70.84)	148 (83.62)
	Other	9 (5.88)	3 (12.5)	12 (6.79)
	Sex n (%)			
	Boy	80 (52.28)	7 (29.17)	80 (52.28)
	Girl	73 (47.72)	17 (70.83)	73 (47.71)
	Level of education *n* (%) Daycare or preschool			
		75 (49.01)	2 (8.33)	77 (43.5)
Child with	Kindergarten	13 (8.5)	0 (0)	13 (7.35)
PKU	Primary school	46 (30.06)	9 (37.50)	55 (31,07)
	Secondary school	19 (12.43)	13 (54.17)	32 (18.08)
	Type of PKU n (%)			
	Mild	13 (8.5)	1 (4.16)	14 (7.9)
	Moderate	20 (13.07)	1 (4.16)	21 (11.87)
	Classical	107 (69.93)	19 (79.2)	126 (71.19)
	Do not know/do not wish to answer	13 (8.5)	3 (12.48)	16 (9.04)

**Table 2:** Factorial structure of questionnaire.

Factor 1: Allowed Foods	Factor 2: Forbidden Foods, Easy to Recognize	Factor 3: Limited Foods, NOT Easy to Recognize
Pepper	Diet Coke	Pumpkin
Pineapple	Nachos	Rice
Canned Fruit	Flour	French Fries
Ketchup	Beans	Roasted Potatoes
Onion	Sunflower seeds	Coconut
Lettuce	Granola type cereal	Green peas
Syrup	Hot chocolate mix	Broccoli
Pickles	Hazelnuts	Avocado
Orange	Beef Bouillon	Artichoke
Watermelon	Egg White	Dark Chocolate
Fresh orange juice Melon Kiwi Olives Plum		Banana

**Table 3:** Goodness of fit statistics.

	*χ* ^2^	NNFI	CFI	GFI	AGFI
Model	912.49 (df = 493)	0.86	0.89	0.99	0.99
Independence model	4324.51 (df = 595)				
Index without diagonal values				0.99	0.98

Note: *χ*^2^ = Chi-Square; df = degrees of freedom; NNFI = non-normalized fit index (Tucker and Lewis, 1973) [36]; CFI = comparative Fit Index; GFI = goodness of fit index; AGFI = adjusted goodness of fit index.

## Data Availability

The data presented in this study are available on request from the corresponding author.

## Supplementary Materials

The following are available online at https://www.mdpi.com/article/10.3390/nu13082719/s1, PKU Questionnaire to Evaluate Knowledge of the Low Phe Diet.
